# Retrospective trial of influence of atrial septal defect closure on manifestation and prognosis of migraine attacks in pediatric patients

**DOI:** 10.1186/1129-2377-14-S1-P101

**Published:** 2013-02-21

**Authors:** YS Lizunou, AS Fedulov, EU Procenko, DB Goncharik, UM Chesnov

**Affiliations:** 1 Belarussian State Medical University, Belarus; 2 Republican Research and Clinical Centre "Cardiology", Belarus

## Introduction

Previous studies found a presence of atrial septal defect (ASD) in some cases of patients with migraine and respective efficacy of ASD closure as a treatment of migraine.

## Purpose

To study an influence of atrial septal defect (ASD) closure on clinical course of migraine in children.

## Methods

Interviewing was conducted in 75 pediatric patients (age 10 to 18 years) operated for ASD (surgical or occluder implantation) in Children's National Cardiology Center «RNPC Cardiology» (n = 75, male 31, female 44, mean age = 15.2 ± 3.4 years) to identify their migraine attacks, migraine specific clinical pictures (evaluation of intensity, location of pain, aura), differentiate migraine pain from other types of headache.

## Results

Before ASD closure migraine was presented in 8 patients (10.67%): 2 males (25%), 6 females (75%). Ñomplete disappearance of migraine after operation was remarked in 2 patients (25%). Reduction of migraine symptoms was found in other 6 patients (75%). Reduction/disappearance of migraine within 1-7 days after ASD closure was observed in 7 patients (87.5%) and within 7 to 14 days in 1 patient (12.5%). Noteworthy, in 6 patients (6.67%) migraine debut was observed in the early postoperative period (after ASD closure). In all 5 patients migraine starts early in postoperative period (1 to 3 days). Migraine was transient in all 5 patients (100%) persisting for 7-14 days to 3.5 years.

## Conclusions

The study indicate that closure of ASD in children effectively reduced migraine in most patients (77.8%), and completely eliminated migraine attacks in other 22.2% of cases, most often in early postoperative period (88.9%). Most often migraine decreased / disappeared in the period 1-14 days after ASD closure. Probability of migraine reduction /disappearance > 14 days after ASD closure is minimal. In some patients (6.8%) debut of migraine was observed early (within first 3 days) in postoperative period but migraine symptom were transient in all patients (100%).
